# *rac*-4*H*,5*H*,6*H*,7*H*,8*H*,9*H*,10*H*,11*H*-Cyclo­deca­[*d*][1,2,3]selena­diazol-4-ol

**DOI:** 10.1107/S2414314626000957

**Published:** 2026-02-05

**Authors:** Dieter Schollmeyer, Heiner Detert

**Affiliations:** a University of Mainz, Department of Chemistry, Duesbergweg 10-14, 55099 Mainz, Germany; Goethe-Universität Frankfurt, Germany

**Keywords:** crystal structure, heterocycle, selenium, medium-sized ring, hydrogen bond

## Abstract

Two mol­ecules of the title compound, C_10_H_16_N_2_OSe, with a chair conformation are connected *via* hydrogen bonds into centrosymmetric dimers. C—H⋯O hydrogen bonds inter­connect the dimers.

## Abstract 2

Mol­ecules of the title compound, C_10_H_16_N_2_OSe, adopt a chair conformation and are connected *via* hydrogen bonds into centrosymmetric dimers. C—H⋯O hydrogen bonds inter­connect the dimers.

## Graphical abstract

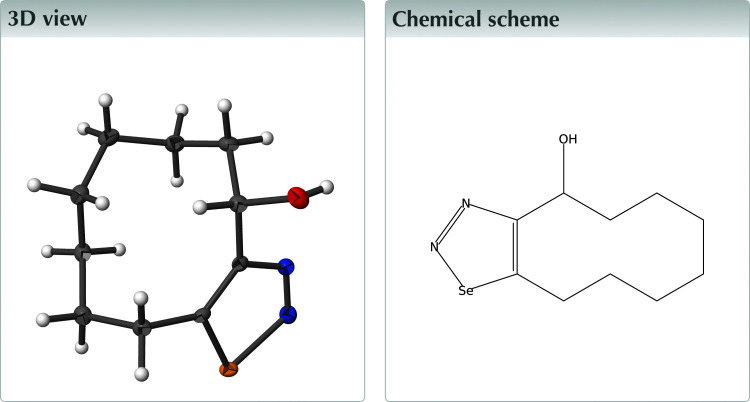

## Structure description

**Figure 1:**
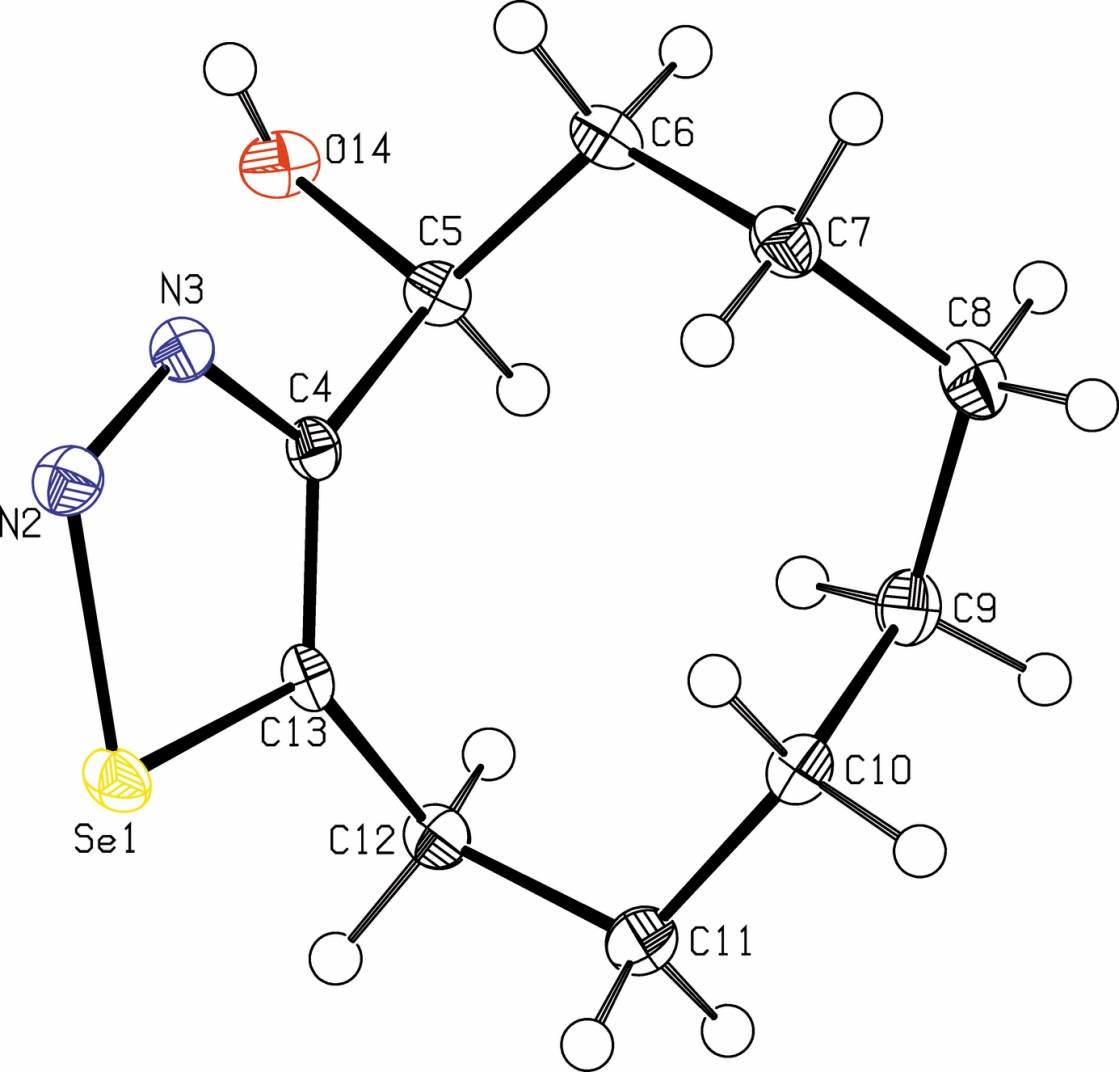
View of the title compound. Displacement ellipsoids are drawn at the 50% probability level.

**Figure 2:**
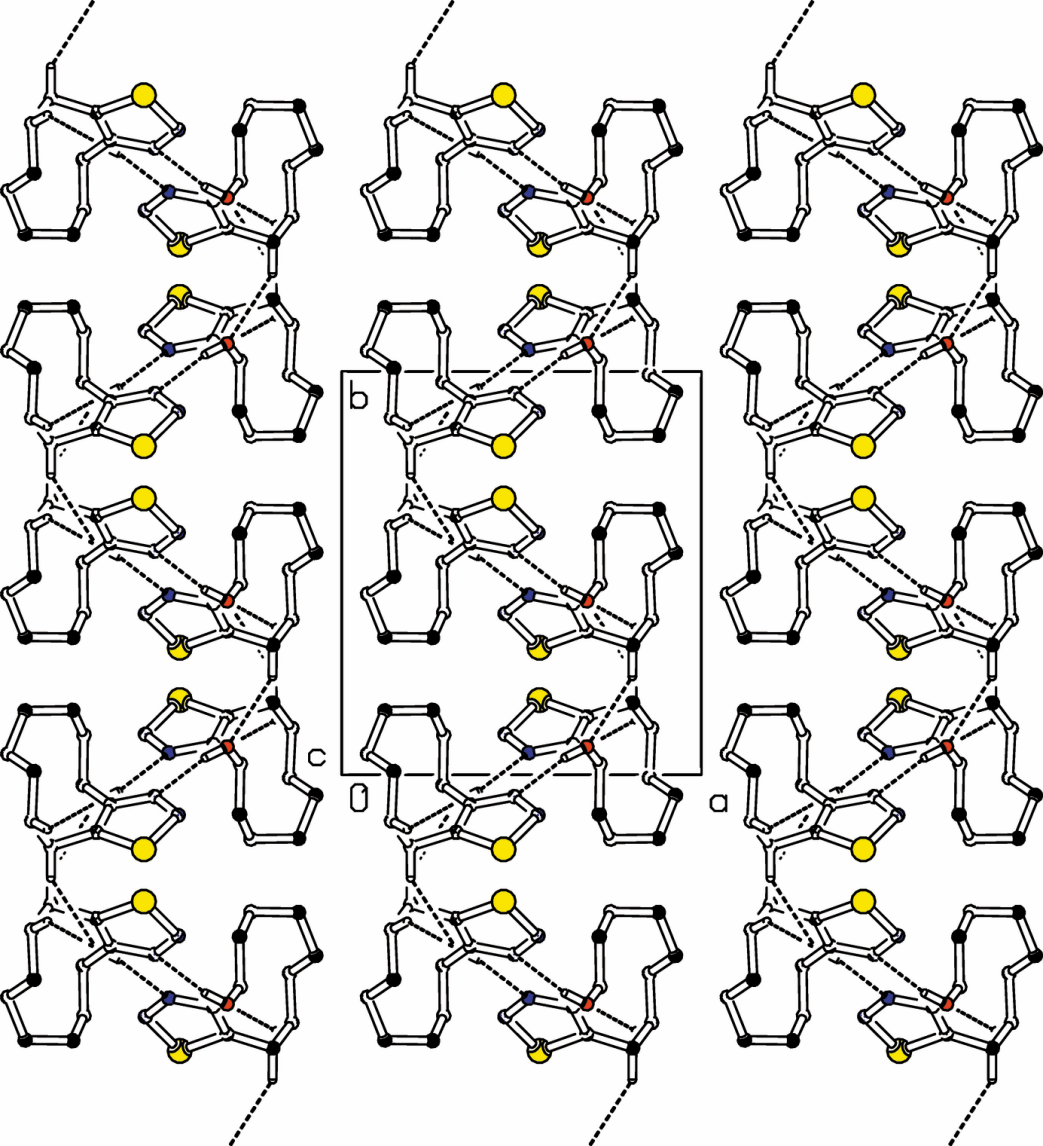
Part of the packing diagram. Hydrogen bonds are drawn with dashed lines. View along *c*-axis direction. Only hydrogen atoms involved in hydrogen bonds are shown for clarity.

The title compound, C_10_H_16_N_2_OSe (Fig. 1 ▸), was prepared in a project focusing on transannular cyclizations (Detert *et al.*, 1992 ▸; Krämer *et al.*, 2009 ▸; Meier *et al.*, ▸) in medium-sized cyclo­alkynes (Detert & Schollmeyer, 2021 ▸; Herges *et al.*, 2005 ▸). The mol­ecule adopts a chair conformation. There are two planes, one is the heterocycle and adjacent C atoms (C5, C12), the other is composed of C5, C6, C7 and C10, C11, C12. The former is planar within 0.0357 (16) Å at C5, the latter within 0.1002 (17) Å at C7. Both planes are close to orthogonal, making a dihedral angle of 85.02 (5)°. The methyl­ene groups CH_2_-8, CH_2_-9 are staggered. In the crystal, pairs of mol­ecules form centrosymmetric dimers, connected *via* O14—H14⋯N3^i^ hydrogen bonds (Table 1 ▸, Fig. 2 ▸). The mol­ecules of the dimers are connected to neighbouring mol­ecules, one *via* a *c*-glide plane, the other one *via* translation along the *c*-axis. For details of the C—H⋯O hydrogen bonds connecting the dimers, see Table 1 ▸.

## Synthesis and crystallization

The sample was prepared from sebacoin (Prelog *et al.*, 1947 ▸; Rühlmann, 1971 ▸) *via* acetyl­ation (Carlson & Bateman 1967 ▸), formation and oxidation of its semicarbazone and de­acetyl­ation with 2-amino­ethanol, m.p. 418 K. Crystallization was by slow evaporation of a solution in methanol/di­chloro­methane. ^1^H-NMR (250 MHz, CDCl_3_): 5.18 (*dd*, 1 H, 4-H), 3.24 (*m*, 2H), 2.18-2.43 (*m*, 3 H), 1.58-1.90 (*m*, 2H), 1.15-1.55 (*m*, 8 H). ^13^C-NMR (100 MHz, CDCl_3_): 161.96 (C-3a, ^2^*J*_C—Se_ = 27 Hz), 161.34 (C-11*a*, ^1^*J*_C—Se_ = 135 Hz), 67.53 (C-4), 38.78 (C-11), 30.32, 26.94, 25.04, 24.21, 21.88, 19.70. ^77^Se-NMR (76.3 MHz, CDCl_3_): 1525.8 p.p.m. (Me_2_Se = 0).

## Refinement

Crystal data, data collection and structure refinement details are summarized in Table 2 ▸.

## Supplementary Material

Crystal structure: contains datablock(s) I, global. DOI: 10.1107/S2414314626000957/bt4197sup1.cif

Structure factors: contains datablock(s) I. DOI: 10.1107/S2414314626000957/bt4197Isup2.hkl

Supporting information file. DOI: 10.1107/S2414314626000957/bt4197Isup3.cml

CCDC reference: 2526821

Additional supporting information:  crystallographic information; 3D view; checkCIF report

Additional supporting information:  crystallographic information; 3D view; checkCIF report

## Figures and Tables

**Table 1:** Hydrogen-bond geometry (Å, °)

*D*—H⋯*A*	*D*—H	H⋯*A*	*D*⋯*A*	*D*—H⋯*A*
C12—H12*B*⋯O14^i^	0.99	2.58	3.430 (2)	144
O14—H14⋯N3^ii^	0.78 (3)	2.22 (3)	2.9801 (18)	165 (2)
C11—H11*A*⋯O14^iii^	0.99	2.56	3.419 (2)	145

Symmetry codes: (i) 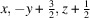; (ii) 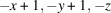; (iii) 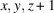.

**Table 2:** Experimental details

Crystal data	
Chemical formula	C_10_H_16_N_2_OSe
*M*_r_	259.21
Crystal system, space group	Monoclinic, *P*2_1_/*c*
Temperature (K)	120
*a*, *b*, *c* (Å)	11.3765 (5), 12.3700 (4), 7.6033 (3)
β (°)	103.747 (3)
*V* (Å^3^)	1039.34 (7)
*Z*	4
Radiation type	Mo *K*α
μ (mm^−1^)	3.58
Crystal size (mm)	0.35 × 0.32 × 0.08

Data collection	
Diffractometer	Stoe Stadivari
Absorption correction	Integration [*X-RED32* (Stoe & Cie, 2020 ▸), absorption correction by Gaussian integration, analogous to Coppens (1970 ▸)]
*T*_min_, *T*_max_	0.672, 0.911
No. of measured, independent and observed [*I* > 2σ(*I*)] reflections	9011, 2636, 2338
*R*_int_	0.023
(sin θ/λ)_max_ (Å^−1^)	0.671

Refinement	
*R*[*F*^2^ > 2σ(*F*^2^)], *wR*(*F*^2^), *S*	0.025, 0.068, 1.08
No. of reflections	2636
No. of parameters	131
H-atom treatment	H atoms treated by a mixture of independent and constrained refinement
Δρ_max_, Δρ_min_ (e Å^−3^)	0.63, −0.43

Computer programs: *X-AREA WinXpose*, *Recipe* and *Integrate* (Stoe & Cie, 2020 ▸), *SHELXT2014* (Sheldrick, 2015*a* ▸), *SHELXL2019/2* (Sheldrick, 2015*b* ▸) and *PLATON* (Spek, 2009 ▸).

## full crystallographic data

### rac-4H,5H,6H,7H,8H,9H,10H,11H-Cyclodeca[d][1,2,3]selenadiazol-4-ol Crystal data

**Table:**

C_10_H_16_N_2_OSe	*D*_x_ = 1.657 Mg m^−^^3^
*M**_r_* = 259.21	Melting point: 418 K
Monoclinic, *P*2_1_/*c*	Mo *K*α radiation, λ = 0.71073 Å
*a* = 11.3765 (5) Å	Cell parameters from 15860 reflections
*b* = 12.3700 (4) Å	θ = 2.5–33.7°
*c* = 7.6033 (3) Å	µ = 3.58 mm^−^^1^
β = 103.747 (3)°	*T* = 120 K
*V* = 1039.34 (7) Å^3^	Plate, brown
*Z* = 4	0.35 × 0.32 × 0.08 mm
*F*(000) = 528	

### rac-4H,5H,6H,7H,8H,9H,10H,11H-Cyclodeca[d][1,2,3]selenadiazol-4-ol Data collection

**Table:**

Stoe Stadivari diffractometer	2636 independent reflections
Radiation source: Axo Mo	2338 reflections with *I* > 2σ(*I*)
Detector resolution: 13.33 pixels mm^-1^	*R*_int_ = 0.023
rotation method, ω scans	θ_max_ = 28.5°, θ_min_ = 2.5°
Absorption correction: integration [X-Red32 (Stoe & Cie, 2020), absorption correction by Gaussian integration, analogous to Coppens (1970)]	*h* = −15→15
*T*_min_ = 0.672, *T*_max_ = 0.911	*k* = −16→16
9011 measured reflections	*l* = −10→10

### rac-4H,5H,6H,7H,8H,9H,10H,11H-Cyclodeca[d][1,2,3]selenadiazol-4-ol Refinement

**Table:**

Refinement on *F*^2^	Primary atom site location: dual
Least-squares matrix: full	Hydrogen site location: mixed
*R*[*F*^2^ > 2σ(*F*^2^)] = 0.025	H atoms treated by a mixture of independent and constrained refinement
*wR*(*F*^2^) = 0.068	*w* = 1/[σ^2^(*F*_o_^2^) + (0.0454*P*)^2^ + 0.0208*P*] where *P* = (*F*_o_^2^ + 2*F*_c_^2^)/3
*S* = 1.08	(Δ/σ)_max_ = 0.001
2636 reflections	Δρ_max_ = 0.63 e Å^−^^3^
131 parameters	Δρ_min_ = −0.43 e Å^−^^3^
0 restraints	

### rac-4H,5H,6H,7H,8H,9H,10H,11H-Cyclodeca[d][1,2,3]selenadiazol-4-ol Special details

**Table:**

Geometry. All esds (except the esd in the dihedral angle between two l.s. planes) are estimated using the full covariance matrix. The cell esds are taken into account individually in the estimation of esds in distances, angles and torsion angles; correlations between esds in cell parameters are only used when they are defined by crystal symmetry. An approximate (isotropic) treatment of cell esds is used for estimating esds involving l.s. planes.
Refinement. Hydrogen atoms attached to carbons were placed at calculated positions and were refined in the riding-model approximation with C_methylene_–H = 0.99 Å, C_tertiary_–H = 1.00 Å, and with *U*_iso_(H) = 1.2 *U*_eq_(C). Hydrogen atom H14 attached to O14 was refined isotropically.

### rac-4H,5H,6H,7H,8H,9H,10H,11H-Cyclodeca[d][1,2,3]selenadiazol-4-ol Fractional atomic coordinates and isotropic or equivalent isotropic displacement parameters (Å^2^)

**Table:**

	*x*	*y*	*z*	*U*_iso_*/*U*_eq_	
Se1	0.45006 (2)	0.68523 (2)	0.48407 (2)	0.01632 (7)	
N2	0.54559 (12)	0.60029 (12)	0.37125 (19)	0.0175 (3)	
N3	0.48019 (12)	0.55443 (11)	0.23022 (18)	0.0148 (3)	
C4	0.35750 (14)	0.57504 (12)	0.1901 (2)	0.0128 (3)	
C5	0.28163 (15)	0.52610 (13)	0.0178 (2)	0.0143 (3)	
H5	0.195907	0.548864	0.007480	0.017*	
C6	0.28386 (16)	0.40234 (13)	0.0134 (2)	0.0179 (3)	
H6A	0.365938	0.379757	0.005564	0.021*	
H6B	0.227068	0.378654	−0.099816	0.021*	
C7	0.25231 (16)	0.34018 (14)	0.1707 (2)	0.0185 (3)	
H7A	0.303042	0.369285	0.285035	0.022*	
H7B	0.276149	0.263773	0.161795	0.022*	
C8	0.12010 (16)	0.34178 (15)	0.1858 (3)	0.0206 (4)	
H8A	0.068247	0.320765	0.066855	0.025*	
H8B	0.110468	0.285582	0.274041	0.025*	
C9	0.07213 (15)	0.44839 (14)	0.2427 (2)	0.0182 (3)	
H9A	−0.012947	0.437897	0.249508	0.022*	
H9B	0.072662	0.503203	0.148010	0.022*	
C10	0.14468 (14)	0.49220 (13)	0.4253 (2)	0.0157 (3)	
H10A	0.113867	0.458365	0.523539	0.019*	
H10B	0.230420	0.470593	0.442049	0.019*	
C11	0.13838 (15)	0.61496 (14)	0.4428 (2)	0.0182 (3)	
H11A	0.182414	0.635982	0.566559	0.022*	
H11B	0.052684	0.636301	0.427624	0.022*	
C12	0.19156 (15)	0.67821 (12)	0.3053 (2)	0.0154 (3)	
H12A	0.139014	0.667721	0.182503	0.018*	
H12B	0.191917	0.756274	0.334065	0.018*	
C13	0.31837 (14)	0.64318 (13)	0.3054 (2)	0.0132 (3)	
O14	0.31845 (12)	0.56888 (10)	−0.13529 (16)	0.0180 (3)	
H14	0.379 (2)	0.542 (2)	−0.143 (3)	0.037 (7)*	

### rac-4H,5H,6H,7H,8H,9H,10H,11H-Cyclodeca[d][1,2,3]selenadiazol-4-ol Atomic displacement parameters (Å^2^)

**Table:**

	*U*^11^	*U*^22^	*U*^33^	*U*^12^	*U*^13^	*U*^23^
Se1	0.01354 (10)	0.01686 (11)	0.01669 (11)	−0.00094 (6)	−0.00010 (7)	−0.00468 (6)
N2	0.0136 (6)	0.0191 (7)	0.0192 (7)	0.0020 (6)	0.0030 (5)	0.0000 (6)
N3	0.0138 (6)	0.0145 (7)	0.0156 (7)	0.0008 (5)	0.0028 (5)	0.0003 (6)
C4	0.0141 (7)	0.0106 (7)	0.0134 (7)	−0.0012 (6)	0.0029 (6)	0.0017 (6)
C5	0.0146 (7)	0.0148 (8)	0.0125 (7)	0.0012 (6)	0.0010 (6)	0.0007 (6)
C6	0.0209 (8)	0.0148 (8)	0.0174 (8)	−0.0005 (7)	0.0033 (7)	−0.0038 (7)
C7	0.0201 (8)	0.0134 (8)	0.0216 (8)	0.0011 (7)	0.0040 (7)	0.0002 (7)
C8	0.0194 (8)	0.0178 (8)	0.0232 (9)	−0.0039 (7)	0.0019 (7)	−0.0023 (7)
C9	0.0148 (7)	0.0197 (8)	0.0196 (8)	−0.0017 (7)	0.0028 (6)	0.0006 (7)
C10	0.0136 (8)	0.0185 (8)	0.0144 (7)	0.0009 (6)	0.0024 (6)	0.0025 (6)
C11	0.0166 (8)	0.0196 (8)	0.0189 (8)	0.0013 (6)	0.0051 (7)	0.0001 (7)
C12	0.0140 (8)	0.0150 (8)	0.0162 (8)	0.0010 (6)	0.0019 (6)	−0.0007 (6)
C13	0.0136 (7)	0.0103 (7)	0.0146 (7)	−0.0027 (6)	0.0012 (6)	0.0003 (6)
O14	0.0214 (6)	0.0183 (6)	0.0147 (6)	0.0046 (5)	0.0048 (5)	0.0027 (5)

### rac-4H,5H,6H,7H,8H,9H,10H,11H-Cyclodeca[d][1,2,3]selenadiazol-4-ol Geometric parameters (Å, º)

**Table:**

Se1—C13	1.8418 (16)	C8—H8A	0.9900
Se1—N2	1.8612 (14)	C8—H8B	0.9900
N2—N3	1.2830 (19)	C9—C10	1.536 (2)
N3—C4	1.380 (2)	C9—H9A	0.9900
C4—C13	1.365 (2)	C9—H9B	0.9900
C4—C5	1.513 (2)	C10—C11	1.527 (2)
C5—O14	1.4294 (19)	C10—H10A	0.9900
C5—C6	1.532 (2)	C10—H10B	0.9900
C5—H5	1.0000	C11—C12	1.540 (2)
C6—C7	1.534 (2)	C11—H11A	0.9900
C6—H6A	0.9900	C11—H11B	0.9900
C6—H6B	0.9900	C12—C13	1.506 (2)
C7—C8	1.536 (2)	C12—H12A	0.9900
C7—H7A	0.9900	C12—H12B	0.9900
C7—H7B	0.9900	O14—H14	0.78 (3)
C8—C9	1.528 (2)		
			
C13—Se1—N2	87.85 (7)	H8A—C8—H8B	107.3
N3—N2—Se1	110.41 (11)	C8—C9—C10	114.14 (14)
N2—N3—C4	117.28 (13)	C8—C9—H9A	108.7
C13—C4—N3	116.05 (14)	C10—C9—H9A	108.7
C13—C4—C5	126.66 (14)	C8—C9—H9B	108.7
N3—C4—C5	117.17 (14)	C10—C9—H9B	108.7
O14—C5—C4	109.85 (13)	H9A—C9—H9B	107.6
O14—C5—C6	110.03 (13)	C11—C10—C9	113.78 (14)
C4—C5—C6	114.20 (13)	C11—C10—H10A	108.8
O14—C5—H5	107.5	C9—C10—H10A	108.8
C4—C5—H5	107.5	C11—C10—H10B	108.8
C6—C5—H5	107.5	C9—C10—H10B	108.8
C5—C6—C7	118.40 (14)	H10A—C10—H10B	107.7
C5—C6—H6A	107.7	C10—C11—C12	114.32 (14)
C7—C6—H6A	107.7	C10—C11—H11A	108.7
C5—C6—H6B	107.7	C12—C11—H11A	108.7
C7—C6—H6B	107.7	C10—C11—H11B	108.7
H6A—C6—H6B	107.1	C12—C11—H11B	108.7
C6—C7—C8	117.81 (15)	H11A—C11—H11B	107.6
C6—C7—H7A	107.9	C13—C12—C11	112.58 (14)
C8—C7—H7A	107.9	C13—C12—H12A	109.1
C6—C7—H7B	107.9	C11—C12—H12A	109.1
C8—C7—H7B	107.9	C13—C12—H12B	109.1
H7A—C7—H7B	107.2	C11—C12—H12B	109.1
C9—C8—C7	117.02 (15)	H12A—C12—H12B	107.8
C9—C8—H8A	108.0	C4—C13—C12	129.46 (14)
C7—C8—H8A	108.0	C4—C13—Se1	108.42 (11)
C9—C8—H8B	108.0	C12—C13—Se1	122.04 (12)
C7—C8—H8B	108.0	C5—O14—H14	109.8 (18)
			
C13—Se1—N2—N3	−0.03 (12)	C7—C8—C9—C10	−57.3 (2)
Se1—N2—N3—C4	−0.13 (17)	C8—C9—C10—C11	153.10 (15)
N2—N3—C4—C13	0.3 (2)	C9—C10—C11—C12	−62.33 (19)
N2—N3—C4—C5	176.55 (14)	C10—C11—C12—C13	−52.79 (19)
C13—C4—C5—O14	113.06 (17)	N3—C4—C13—C12	−177.09 (15)
N3—C4—C5—O14	−62.74 (18)	C5—C4—C13—C12	7.1 (3)
C13—C4—C5—C6	−122.78 (18)	N3—C4—C13—Se1	−0.29 (17)
N3—C4—C5—C6	61.42 (18)	C5—C4—C13—Se1	−176.14 (13)
O14—C5—C6—C7	177.50 (14)	C11—C12—C13—C4	99.6 (2)
C4—C5—C6—C7	53.4 (2)	C11—C12—C13—Se1	−76.78 (16)
C5—C6—C7—C8	70.6 (2)	N2—Se1—C13—C4	0.18 (12)
C6—C7—C8—C9	−70.8 (2)	N2—Se1—C13—C12	177.26 (14)

### rac-4H,5H,6H,7H,8H,9H,10H,11H-Cyclodeca[d][1,2,3]selenadiazol-4-ol Hydrogen-bond geometry (Å, º)

**Table:**

*D*—H···*A*	*D*—H	H···*A*	*D*···*A*	*D*—H···*A*
C12—H12*B*···O14^i^	0.99	2.58	3.430 (2)	144
O14—H14···N3^ii^	0.78 (3)	2.22 (3)	2.9801 (18)	165 (2)
C11—H11*A*···O14^iii^	0.99	2.56	3.419 (2)	145

Symmetry codes: (i) *x*, −*y*+3/2, *z*+1/2; (ii) −*x*+1, −*y*+1, −*z*; (iii) *x*, *y*, *z*+1.
